# Nanofiber-Enhanced “Lucky-Bag” Triboelectric Nanogenerator for Efficient Wave Energy Harvesting by Soft-Contact Structure

**DOI:** 10.3390/nano12162792

**Published:** 2022-08-15

**Authors:** Yuanzheng Luo, Buyin Li, Lianghao Mo, Zhicheng Ye, Haonan Shen, Yuan Lu, Shufa Li

**Affiliations:** 1 School of Electronic Information Engineering, Guangdong Ocean University, Zhanjiang 524088, China; 2 School of Optical and Electronic Information, Huazhong University of Science and Technology, Wuhan 430074, China

**Keywords:** triboelectric nanogenerator, nanocellulose, wave energy

## Abstract

Developing clean and renewable ocean wave energy is a top priority and an effective way to achieve carbon neutrality. Triboelectric nanogenerators (TENGs) have emerged as promising green and clean energy-harvesting devices. To harvest low-frequency wave energy efficiently, much effort has been made on the modification of the contact surface, which leads to a higher fabrication cost. In this work, we designed a novel “Lucky-Bag” core (LBC) for spherical TENGs with a low-cost and easy fabricating process. The nanofiber/silicone hybrid porous outer layer of the LBC can switch freely from plane to surface and improve the output performance of both the plane and spherical TENGs. Several factors, such as the input frequency, direction, and resistive load, together with the thickness were systematically investigated; the unique porous soft-contact structure increased the triboelectric contact area, and the working mechanism was studied by using the COMSOL software. The experimental results showed that the peak-to-peak open-circuit voltage (V${}_{oc}$) and short-circuit current (I${}_{sc}$) could reach 580 V and 23.5 μA at 1.5 Hz, even under 2D linear motion. Besides, the maximum output power of the spherical TENGs reached 9.10 mW, which can fully power electronic devices such as capacitors and LEDs under water wave triggering. These findings provide useful guidance for optimizing the performance of spherical TENGs for practical applications in harvesting water wave energy.

## 1. Introduction

Harnessing natural forces to provide energy to humans is as old as the first sailing ship, driving people to urgently search for new energy sources in our environment. For decades, renewable and clean energy technologies have provided a rapidly increasing share of the energy used in the whole world. Although solar power, wind power, water power, and other technical fields have seen substantial cost declines, climate change is dramatically impacting the long-term sustainability of these ambient energy collection technologies [1]. The ocean occupies 71% of the world’s area, and the global wave energy reserves are estimated to be more than 2TW globally [2]. Such abundant blue energy can achieve carbon neutrality with superior advantages with little dependence on the environmental conditions [3]. Ocean waves exhibit rather low frequencies (less than 2 Hz), yet the classic electromagnetic generator (EMG) performs relatively poorly in low-frequency energy harvesting [4,5]. On the other hand, the traditional water flow power generator is also limited by high cost, low reliability, and other engineering challenges related to installing devices to convert blue energy [6]. Therefore, it remains a challenge to harvest the abundant renewable ocean wave energy on the Earth’s surface with new technologies and devices.

Since first being reported in 2012, triboelectric nanogenerators (TENGs) have shown great potential for macro-scale wave energy harvesting with outstanding characteristics, including high energy conversion efficiency, an ultrahigh output voltage, abundant material sources, etc. [7,8]. Among four basic operating modes, the most classical contact-separation (CS) mode is easily constructed, and it could harvest energy from various water-related sources, such as rain [9], water flow [10], etc. However, the low-frequency wave motion hardly triggers energy harvesting in the CS capacitor mode with two parallel plates [11]. Inversely, TENGs with other bending structures were adopted to directly harvest the impact energy from water waves, such as arch-shaped [12] and wavy electrodes [13]. Jiang et al. designed an arch-shaped TENG using indium tin oxide (ITO) and polytetrafluoroethene (PTFE) film, which was supported by substrates to directly harvest the wave impact energy with an excellent output current of 4.8 mA [14]. As elaborated in a previous review [2], most plane-structured TENGs can obtain higher I${}_{sc}$ output and power density than rolling-spherical-structured TENGs. Meanwhile, some rolling-structured TENGs based on the freestanding (FS) mechanism are designed to convert the lower and more unstable wave energy via the freestanding balls, such as PTFE ball [15],silicone rubber ball [16], PDMS ball [17] and etc. [18]. Wang firstly proposed a rolling spherical structure containing a solid Nylon ball inside, in which the Nylon balls rub against the Kapton friction layer, resulting in electron transfer between interdigitated electrodes [19]. Cheng et al. considered the influence of the ball hardness and proposed soft silicone liquid balls to replace the traditional hard-contact structure [20]. The output of the soft-contact spherical TENGs is tunable by controlling the softness between friction layers. Han et al. designed a soft-triggering rotational TENG to provide controllable polarity advancement with high DC power density (32 mW m${}^{-2}$) and long-term durability [21]. Briefly, the contact material systems of the CS and FS modes commonly have functional differences; their electron-donating ability, surface area, and nanoscale roughness are often incompatible [22,23]. The various morphologies/surface engineering used for complex nanostructure fabrications of the CS mode film are not applied to the FS mode, especially not being suitable for harvesting the low-frequency and unstable wave energy. Thus, transferring the high outputs of the planar CS mode TENGs to other modes is still a bottleneck.

Among a wide range of triboelectric polymers (artificial and natural), silicone rubber and cellulose nanofiber are widely used in high-performance TENGs [24,25,26]. The triboelectric charge density of food-grade silicone rubber could reach around 100 μC m${}^{-2}$ on the surface [27]. The cellulose-based planar TENGs can reach a maximum peak of 300 Wm${}^{-2}$, which is a new record for green-material-based TENGs [28]. Additionally, some rolling-friction-based TENGs with a silicone–elastic-based layer structure were reported to achieve a high output performance. Xu [22] constructed a high-performance TENG using treated silicone rubber as the triboelectric material. Under low-frequency sinusoidal trigger, the peak power of one TENG unit can reach 1 mW and 1.28 mW for agitations of 3 Hz and 5 Hz, respectively. Meanwhile, various microstructure and nanostructure surface engineering has been conducted to enhance the contact triboelectrification of the electrification process [29], such as plasma treatment [30], UV irradiation [31], nanoparticle decoration [32,33] etc. Although these microscale/nanoscale modifications on thin films are critical to ensure the high-performance applications of planar TENGs, for nonplanar and multilayer-structured TENGs, these approaches involve a complex, expensive, and time-consuming preparation. Therefore, a more compatible and effective technique to fabricate a universal friction layer suitable for both planar and nonplanar TENG devices remains to be explored.

Herein, we report a novel “Luck-Bag” prototype of an optimized soft-contact structure driven by a nanofiber-enhanced spherical TENG (NS-TENG). The porous surface uses nanocrystal-cellulose (CNC)-based silicone film and commercial Kapton as the tribo-layers and aluminum foil as the electrodes. The hollow core coating silicone replaces the traditional hard/soft ball with a porous CNC/silicone layer. The porous elastic layer enlarges the contact area and results in a three-times enhancement in the performance. Under the 2 Hz x-y cross-motion triggering, the NS-TENG can deliver a peak power of 9.1 mW and a short-circuit current of 23.5 μA, which is comparable to some spherical TENGs with multilayered structures and unique surface engineering. Moreover, the NS-TENG array can be used for continuously powering light-emitting diode (LED) lamps and easy triggering by wave energy. By utilizing the eco-friendliness, foldability, and easy fabrication, the NS-TENG displays the potential for large-scale blue energy harvesting.

## 2. Materials and Methods

### 2.1. Materials

The cellulose nanocrystals were obtained from ScienceK (sciencek.com). The commercial CNCs with a cellulose Iβ crystalline structure were prepared by H${}_{2}$SO${}_{4}$ hydrolysis of native wood, and the diameter and length of the CNC were around 2.3–4.5 nm and 44–108 nm. The nanostructured cellulose fibers (nanofibers) derived from plants are generally categorized into nanocrystalline cellulose (or cellulose nanocrystals) and cellulose nanofibrils, and the CNCs used in our experiment belong to the former. The polydimethylsiloxane (PDMS) silicone elastomer kit (Sylgard 184), containing the polymeric base/curing agent in a mass ratio of 10:1, was purchased from Dow Corning. The Ecoflex 00-30 silicone rubber of hardness Shore 00-30 was obtained from Smooth-On (smooth-on.com). The hollow acrylic sphere and Al electrode were bought from a local hardware store. All the other reagents were used as received without treatment.

### 2.2. Fabrication of the Plane TENG

We fabricated plane TENGs by using the CNC nanofiber-enhanced silicone polymer of a 15 wt% concentration as a friction layer and prepared two more friction layers for comparison. Firstly, the commercial nanofiber powder (CNC) was added to the silicone bases (Ecoflex/PDMS) using a magnetic stirrer at room temperature. The concentration of CNCs varied from 5 wt% to 20 wt% with an interval of 5 wt%, and obvious agglomeration was observed when the amount of CNCs used was over 15 wt%. Thus, in subsequent experiments, mixtures loaded with 15 wt% of nanocellulose additive were prepared. After being mixed evenly with the curing component, the liquid CNC/polymer precursor was poured into a rectangular groove mold and cured with fine granulated sugar at a ratio of 35:1. Subsequently, we performed a drying procedure in an air-circulating oven at 90 °C for 30 min. This step is conducive to the complete penetration of the PDMS mixture into the gap of the nanoparticlesand fully exhausts the air bubbles. A water bath was then used to remove the sugar template and obtain the rectangular nanofiber-enhanced silicone porous (NSP) film. Finally, the plane TENG was assembled by using a polyimide (PI) film as a collector and copper-plated electrodes on both sides of the printed circuit board sheet as the electrode disks (5 mm in thickness). Two lead wires were connected, respectively, to the two sets of electrodes.

### 2.3. Fabrication of the NS-TENG

**Figure 1:**
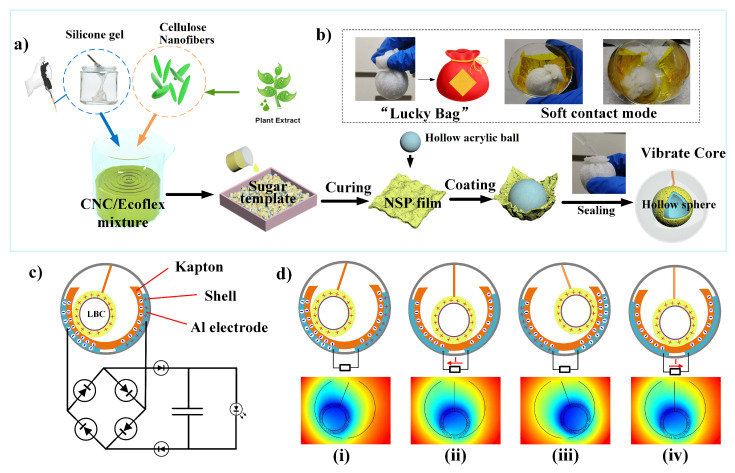
(**a**) Schematic illustration for the synthesis of the NSP film and assembly of the rolling vibrator; (**b**–**d**) Corresponding digital photographs of the L-ball and NS-TENG. (**c**) Circuit diagram of the self-powered system consisting of a TENG and a capacitor. (**d**) Charge distribution scheme of the device under the short-circuit condition and the corresponding simulated potential distributions under the open-circuit condition.

The “Luck-Bag” vibration core (LBC) was prepared through a straightforward process by one-step warping of the NSP film, specifically, taking an NSP film and folding the outer layer to the middle in a circle to make a round wrapper. Top with a hollow acrylic ball (6 cm in diameter) of filling, and bring the sides up to cover the filling like a petal around the hollow ball. Tie the open side with a self-locking strip to make a pouch, and seal the hole with liquid silicone via curing progress. Finally, the “Luck-Bag” vibration cores of different coating films were prepared with PDMS, porous PDMS, Ecoflex, and porous Ecoflex, denoted as PDMS@LBC, P-PDMS@LBC, Eco@LBC, and P-Eco@LBC. The spherical TENG was assembled by using a transparent acrylic spherical shell, Kapton film, and an aluminum electrode, as shown in Figure 1a. A lightweight TENG array device was obtained by connecting 4 NS-TENGs in parallel and encapsulating them in a mold after waterproofing treatment (Figure S1). In this paper, the NSP film, in particular, refers to the film made with Ecoflex as a silicone substrate. Briefly, the porous silicon film was prepared using only three food-grade additives: Ecoflex, cellulose, and sugar. The by-product of the whole preparation process is only recyclable sugar water, realizing green and pollution-free production. The cost-effective method and readily available materials result in a production cost of less than $1 for the entire NS-TENG.

### 2.4. Materials’ Characterization

The current, transferred charges, and capacitor voltage were measured by an electrometer (Keithley 6514). The open-circuit voltage was measured using an electrostatic voltmeter (Trek 347). A step motor imposed the displacement with adjustable speed by a pulse drive circuit. A universal material testing machine that uses a stretch control mode with a stretch strain rate of 100% min${}^{-1}$ was used to provide the required pressure. The simulation of the charge and pressure around the “Luck-Bag” vibration coreunit was based on the commercial software COMSOL. The morphologies and microstructures of the samples were observed using a Hitachi (SU8010) scanning electron microscopy. The water contact angle (CA) was measured by a DSA30 Drop Shape Analyzer (Kruss).

## 3. Results

### 3.1. Structure and Working Principle of NS-TENG

The nanofiber-enhanced spherical triboelectric nanogenerator (NS-TENG) consists of a CNC/ecoflex-layer-coated vibration core and two semi-spherical shells with Al electrodes and dielectric Kapton layers on their inner surfaces. As shown in Figure 1a, the vibration ball was fabricated as a one-step envelope into a lucky-bag with a rectangular NSP film. The thickness of the nanofiber-enhanced silicone layer is far smaller than its plane size. The planar silicone rubber is ideal for forming the spherical coating. For that, its roughness and softness would enhance the actual contact area and contribute to the device’s durability. Unlike the roll motion where the ball rolls along the wave agitation direction, the lucky-bag ball dangled by a string in an unstable state would roll chaotically and can also roll in the perpendicular agitation direction. A photograph of the as-fabricated “Lucky-Bag” ball (L-ball) unit with a dangling structure is shown in Figure 1b and illustrates its detailed structure. The tension of the rope counteracts some of the gravity, while using the tangential force to provide periodic impact to the rigid shell. Figure 1c is the equivalent circuit of the self-powered system consisting of the TENG and a supercapacitor. The type of pair for the TENG is the dielectric-on-dielectric mode. The sign of the triboelectric charges is relative to the counterpart, and the two friction layers (NSP film and PI) that rub against each other have an evident electronegativity difference [27]. Figure 1d shows the operating principle of the NS-TENG. The vibration core rolls back and forth in a spherical shell, in contact with the Kapton film attached to the inner Al foil electrode. When the freestanding L-ball rolls to the left-hand electrode (LE), equal amounts of charges are generated on the top surface of the right Kapton film and the surface of the L-ball (Figure 1d(i)). When the L-ball rolls toward the other end and separates from the initial Kapton surface, the electrons transfer from the left to the right via the external load due to the electrostatic induction process (Figure 1d(ii)). Once the L-ball reaches the overlapping position of the right-hand electrode (RE), the electrons will then be driven to the RE (Figure 1d(iii)). Finally, when the L-ball moves right, electrons start their reverse transfer in the external circuit (Figure 1d(iii)). Therefore, a cyclic periodic electrical signal can be generated based on the conjugation of the triboelectric effect and electrostatic induction.

### 3.2. Surface Characterization of NSP Film

**Figure 2:**
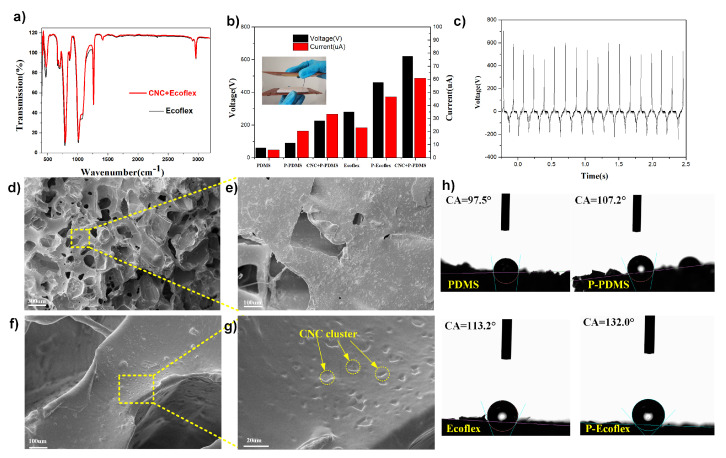
(**a**) FTIR spectra of the nanofiber-enhanced Eco and the original P-Eco samples; (**b**) histogram comparison of different dielectric layer materials; (**c**) voltage output waveform of the CNC/P-Eco layer; (**d**–**g**) corresponding SEM images of the CNC/P-Eco sample; (**h**) the water contact angle of various silicone films.

The Fourier transform infrared (FTIR) spectra were used to evaluate changes in the molecular structure and functional groups of the original and the nanofiber-enhanced silicone rubber (Figure 2a). It can be observed that the FTIR spectra of both the Eco30 and CNC/Eco30 silicone samples showed the same peaks, indicating that the nanofiber adding processes did not alter the chemical composition of the Ecoflex silicone. The enhanced Si-OH signals (at 1430 cm${}^{-1}$) of the CNC/Ecoflex were associated with CH${}_{2}$ scissoring motion and symmetrical bending in cellulose I. Other peaks represent the characteristic peaks of silicone rubber. In the plane TENG structure, a gap allows successive contact and separation under periodic compressive force manually. In addition, pure PDMS, porous PDMS (P-PDMS), CNC/P-PDMS, pure Ecoflex, porous Ecoflex (P-Eco), and CNC/P-Eco sheets with the same thickness as the plane dielectric layer were utilized for the TENG device for comparison. According to the average output voltage, it can quickly filter and compare the suitable triboelectric layer material. Figure 2b shows the TENG’s open-circuit voltage (V${}_{oc}$) with a series of polymer composites. As expected, the flat films without pores produced a very low output voltage with an average value of 30 V. With the successful introduction of pores and nanofiber into the PDMS elastomers, the output performance of the P-TENGs notably increased. In particular, CNC/P-Eco sponges produced V${}_{oc}$ with an average value of 680 V, which was 20-fold enhanced compared to PDMS (Figure 2c). As shown in Figure 2d,e), pores (300–500 μm) can be discernible from the interconnected spongy network. The porous sponge structure allows for more significant deformation on the rolling surface. In the SEM image in Figure 2f,g), the cluster structure of the CNCs was formed on the pore skeleton, which is crucial for the performance of the TENG unit agitated by water waves. We also examined the contact angle of water droplets on various silicone films, as shown in Figure 2h. The stretchable and porous P-Eco film had a water contact angle (WCA) of 132° due to the increased surface roughness, which is much higher than other silicone films. Its softness and roughness would enhance the actual contact area and contribute to the accumulation of electric charges. These unique microstructures of the CNC/P-Eco composites make the dielectric layer surface less sticky and enable a smooth roll of the ball on the dielectric layer with a relatively low-frequency force.

### 3.3. Electric Output Performance of NS-TENG

**Figure 3:**
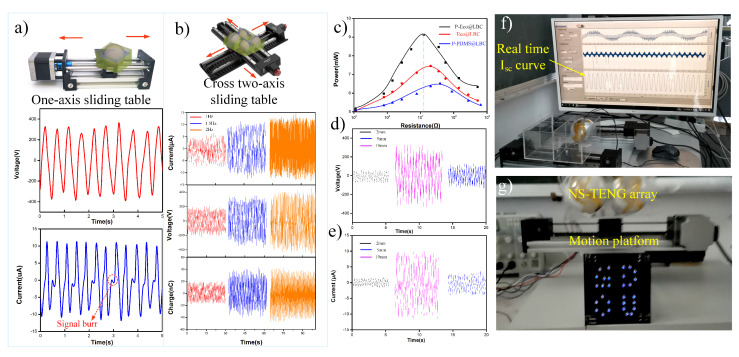
Output performance characterization of NS-TENGs. (**a**) Single guide rail and stepper motor drive setup and outputs of the NS-TENG at 2 Hz. (**b**) The NS-TENG array (2 × 2) experimental setup and outputs at various frequencies. (**c**) The output power comparison of the NS-TENG with various cores at 2 Hz (**d**,**e**). The electrical output comparisons between various thicknesses of the LBC’s coating film at 2 Hz. (**f**) Demonstrations of the NS-TENG’s electric outputs. (**g**) Photograph of the 2 × 2 NS-TENG array as a power source to light the LEDs in the daytime.

Among various solid point-contact models, the most beneficial way to improve the outputs is typically by increasing the movement displacement and frequency in a single direction [19,20]. However, previous studies have found that the current increases monotonically with the frequency from 1–5 Hz and enters an unstable region after 3 Hz [22]. In the actual continuous undulating sea state, the horizontal distance and frequency of movement are usually low due to the action of inertia, and the impact is not in a single direction. Therefore, to comprehensively understand the behavior of the NS-TENG under low frequency with external mechanical excitations, Figure 3 illustrates the electrical output of the NS-TENGs working at a different frequency from 1 Hz to 2 Hz. The schematic diagram of the experimental setup is shown in Figure 3a,b; an x-y linear guideway was utilized to simulate natural ocean waves and provide the impact agitation in a different direction. The LED array can be easily lit by single NS-TENG as shown in Video S1. The displacement amplifier was 20 mm, and the orthogonal direction has a sign on the sketch map. Considering a single NS-TENG, the outputs of the short open-circuit voltage (V${}_{oc}$) and the short-circuit current (I${}_{sc}$) at 1.5 Hz are shown in Figure 3a. The maximum V${}_{sc}$ and I${}_{sc}$ of the NS-TENG can reach around 580 V and 23.5 μA, respectively. Periodic burrs with a small amplitude of driving motion appeared in the Isc curve. This is due to the single pendulum cycle: the ball has two opportunities to move back and forth to the upper electrode, producing two pulse outputs [22]. Notably, the stable output characteristics enable a single NS-TENG to light 28 LEDs dimly (Figure S2). Meanwhile, the electrical output performances of the NS-TENG array (2 × 2) with the increasing movement frequency of the x-y stepping motor are shown in Figure 3b. The results show that the outputs of the NS-TENG array with P-Eco@LBC were stable even on the cross-rail, except that the V${}_{sc}$ and I${}_{sc}$ increased with the higher motion frequency of the stepping motor. From 1 Hz to 2 Hz, its V${}_{sc}$ increased about 196% from 396 V to 780 V, and I${}_{sc}$ increased from 10.8 μA to 29.7 μA. Its maximum transferred charges (Q${}_{sc}$) can reach 76 nC at 2 Hz. Meanwhile, the output power was also affected by external resistance. As shown in Figure 3c, the P-PDMS@LBC, Eco@LBC, and P-Eco@LBC assembled NS-TENGs were measured with a varying load resistance, where the proper match of them releases the maximum peak power output of P-Eco@LBC at 9.1 mW when the external load resistance is 27 M Ohm for agitations of 1.5 Hz. The higher output power is mainly due to the porous soft-contact interface between the core and shell in the spherical TENG, enhancing the effectiveness of the contact between the two triboelectric surfaces, and the surface modification of the nanofibers also influenced the maximum peak power. Additionally, in order to study the effect of the different softness of Eco@LBC on the output, several spherical cores with different coating thicknesses were fabricated; the corresponding outputs are shown in Figure 3d,e. The as-fabricated single NS-TENG converts frictional energy into electricity by producing alternating currents, and Figure 3f shows the stability and persistence of the current curves driven by linear low-frequency motion (Supplementary Video S2). The 2 × 2 array worked as a power source capable of lighting LED array (rated power, 60 mW) in the daytime as illustrated in Figure 3g and Supplementary Videos S3 and S4. Due to the triboelectric nanogenerator converts frictional energy into electricity by producing alternating currents, the LEDs were lit alternately. The high output performance of NS-TENG is also comparable to spherical TENGs with multilayered structures and unique surface engineering as show in Table S1.

**Figure 4:**
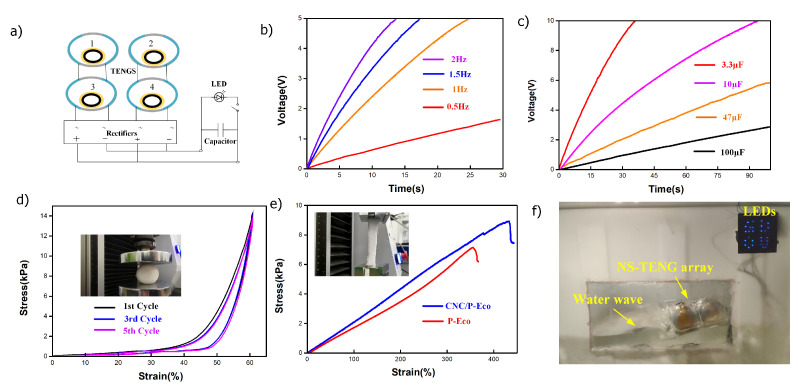
(**a**) Circuit diagram of the self-powered LED circuit consisting of an NS-TENG array and a capacitor; (**b**) charging of 3.3 μF capacitors with different frequencies; (**c**) the charging curves of NS-TENG-2 at 2.0 Hz for different capacitors (3.3 μF, 10 μF, 47 μF, 100 μF) (**d**,**e**); the compressibility and stretchability of the NSP film; (**f**) photo of the NS-TENG array as a power source to light the “GDOU” LED pattern by water wave triggering.

The capacitor-charging characteristics of the NS-TENG were analyzed by using a circuit consisting of a bridge rectifier and various capacitors, as shown in Figure 4a. For all capacitances, the voltage curves rapidly increased in the initial period and then gradually stabilized, implying that the pulse output was transferred into the stable direct current (DC) output.The photograph of NS-TENG charging the capacitor in real time is shown in Figure S3. Figure 4b depicts that the voltages of the 3.3 μF capacitor can be charged to around 5 V by the NS-TENGs in 25 s in a low-frequency range (1–2 Hz). The charging performances of the TENG array with respect to various capacitors from 3.3 μF to 100 μF in 100 s are presented in Figure 4b,c). It only took 90 s for the 2 × 2 NS-TENG array to charge the 10 μF capacitor from 0 to 10 V. The softness of the core surface ensures the NS-TENG can efficiently work with various frequencies of impact force. Thus, the mechanical performance of the NSP film under the compressive and tensile state was further investigated. Axial compression testing of the P-Eco sheet with a density of 35 mgcm${}^{-3}$ revealed its good rebound resilience, and it showed it to be reduplicative compressive, as shown in Figure 4d. In the first cycle (black curve), the stress abruptly increased after 40% elastic strain changes. The porous structure’s elastic bending and shearing deformation provide the initial compressive strength. The stress increased linearly because of the increasing compaction of the polymer network. As cycling continued, the stress path was repeated well, indicating that the soft-contact model was stable and highly efficient. Figure 4e shows that the NSP film was stretched to 430% compared with the original porous Ecoflex sheet without nanofiber additives (380%), presenting excellent stretchability (Figure S4). In the water tank experiment, the output of the 2 × 2 NS-TENG array was rectified, then connected in parallel together to light the 28 LEDs (Figure 4f, Supplementary Video S5).

**Figure 5:**
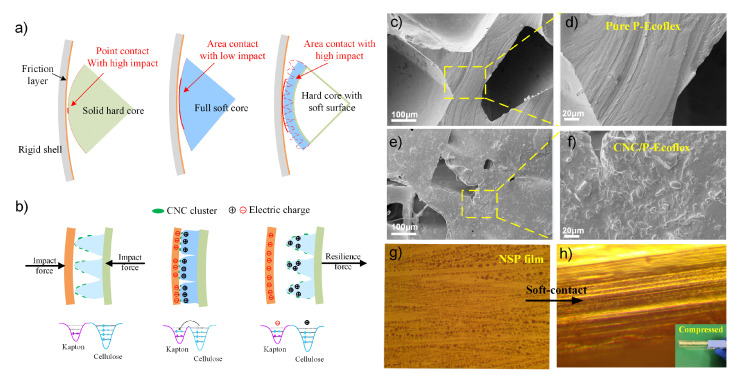
(**a**) Partial enlargement schematic diagram and comparison of the contact modes for other typical rolling spherical TENGs; (**b**) the triboelectrification of the cellulose-fiber-enhanced core before and in contact with the Kapton friction layer based on soft contacting; (**c**–**f**) SEM images of the surface of CNC/P-Ecoflex; (**g**,**h**) Optical micrographs of the NSP film soft-contact process. (The magnification is 1000).

The electric outputs were mainly related to the contact frequency and triboelectric area for the rolling-friction-based TENG. The vital distinction in the contact modes between the NS-TENG and typical TENGs provides insight into the low-frequency output performance. The hard-contact between the core and shell has a high impact force, but the strain of the solid core surface is minor, as shown in Figure 5a. This construction close to point-contact mode results in relatively lower outputs on account of the smaller contact area. The limited fiction area for charge storage takes a long time to accumulate and release the charge. In contrast, the soft-contact with a large triboelectric area can quickly accumulate charge. However, due to insufficient impact strength, it cannot adapt to the low-frequency discharge process. For the NS-TENG, the more appropriate compromise in the middle of hard- and soft-contact is adding a thin and soft NSP film on the surface of the solid sphere, which increases the contact area and maintains the impact force, especially at a low frequency. On the other hand, the high performance of the nanofiber-modified TENG was attributed to the large deformability of the NSP film and refers to the surface roughness provided by the decorated CNC cluster, which enables soft contacting, as shown in Figure 5b. Therefore, the triboelectric effects of the porous-cellulose-fiber-enhanced core are stronger than the other two models, which can be made softer and more rough. Figure 5b also shows a schematic illustration of an electron potential well model of the difference between cellulose fibers and Kapton. Their electrons are tightly confined in their original orbits before contact, due to the high escaping energy barrier. With the impact of mechanical forces, their electron clouds overlap strongly in contact with each other, leading to the significant reduction of the interatomic potential barrier. Eventually, some electrons in the nanocellulose with higher energy can easily overcome the reduced potential barrier and transfer to the Kapton to achieve equilibrium. After the two materials are separated, the transferred electrons and holes remain as static charges on the surfaces of the Kapton and cellulose fibers, respectively. We also compared the amount of charge output between different contact modes, the higher charge levels of soft-contact surface state could store a higher amount of charge than the others as shown in Figure S5. One of the keys to improving the output performance of the TENG is how to increase the effective contact area, which strongly depends on the surface morphology of the friction layer. The traditional morphologies of TENGs, including domes and columns, are generally fabricated using expensive and time-consuming lithography and etching techniques. As shown in Figure 5c,d, the porous silicon structure (pure P-Ecoflex) contains discrete micropores with smooth walls and surfaces, which is unsuitable for the above surface engineering technique. For comparison, the uniform clusters’ surface morphology of the as-obtained CNC/P-Ecoflex endows a higher contact area, as shown in Figure 5e,f. The corresponding NSP film compression process is also shown in the optical micrographs (Figure 5g,h). The combination of nanocellulose-based polymer hybrids and a soft-triggering strategy provides an essential advancement in the output performance of the S-TENG at low operating frequencies

## 4. Conclusions

In conclusion, we presented a nanofiber-enhanced spherical triboelectric nanogenerator (NS-TENG) with a soft-contact structure, which enlarges the contact area and greatly improves the output performance at a low frequency. A single NS-TENG’s peak-to-peak open-circuit voltage (V${}_{oc}$) and short-circuit current (I${}_{sc}$) could reach 580 V and 23.5 μA, driven by a bidirectional force (x-y) at 1.5 Hz. Notably, its unique structure and simplified production process can be tuned to match other types of natural or bio-sourced templates. Our finding provides a cost-effective optimization methodology for spherical TENGs and exhibits their exciting applications in the fields of harvesting large-scale blue energy.

## Data Availability

Not applicable.

## Supplementary Materials

The following Supporting Information can be downloaded at: https://www.mdpi.com/article/10.3390/nano12162792/s1, Figure S1: The photograph of light weight NS-TENG array (200 g); Figure S2: The photograph of single NS-TENG lighted up LED arrays (Video S1); Figure S3: The photograph of the NS-TENG charging a capacitor; Figure S4: Photos of the NSP film (P-Eco) stretching process; Figure S5: (a) schematic diagram of three different contact modes; (b,c) The comparison of output charge curves; Table S1: The output performance comparison of rolling spherical structured TENG; Video S1: LEDs were lightened by one NS-TENG under two-dimensional linear motions at 2 Hz; Video S2: The stable short-circuit current of NS-TENG under various low frequency (1 Hz, 1.5 Hz, 2 Hz); Video S3: 2 × 2 NS-TENG array as a power source to light the LEDs in the daytime; Video S4: 2 × 2 NS-TENG array powering about 100 LEDs in the daytime; Video S5: The NS-TENG array lightens up 28 blue LEDs (60 mW) under the slow water wave motion. References [15,16,17,18,19,20,22] are cited in the supplementary materials.

## Abbreviations

The following abbreviations are used in this manuscript:

TENG	triboelectric nanogenerator
LBC	“Lucky-Bag” core
CNC	nanocrystal cellulose
EMG	electromagnetic generator
NS-TENG	nanofiber-enhanced spherical TENG
