# Supplementary material for: Nanofiber-Enhanced “Lucky-Bag” Triboelectric Nanogenerator for Efficient Wave Energy Harvesting by Soft-Contact Structure
Source: Nanomaterials (Basel). 2022 Aug 15;12(16):2792. doi: 10.3390/nano12162792 (PMC9415517; doi:10.3390/nano12162792)
Supplement: Supplementary file 1 [file nanomaterials-12-02792-s001.zip › nanomaterials-1811559-Supplementary Materials.pdf]

Supplementary Material

Nanofiber Enhanced "Lucky-bag" TENG for Efficient Water Wave Energy Harvesting by Soft Contacted Structure

Yuanzheng Luo [1,†], Buyin Li [2,†], Lianghao Mo [1,†], Zhicheng Ye [1,†], Haonan Shen [1,†], Yuan Lu [1,†] and Shufa Li [1,*]

[1] School of Electronic Information Engineering, Guangdong Ocean University, Zhanjiang 524088, China
[2] School of Optical and Electronic Information, Huazhong University of Science and Technology, Wuhan 430074, China
* Correspondence: Correspondence: lishufa@gdou.edu.cn
† These authors contributed equally to this work

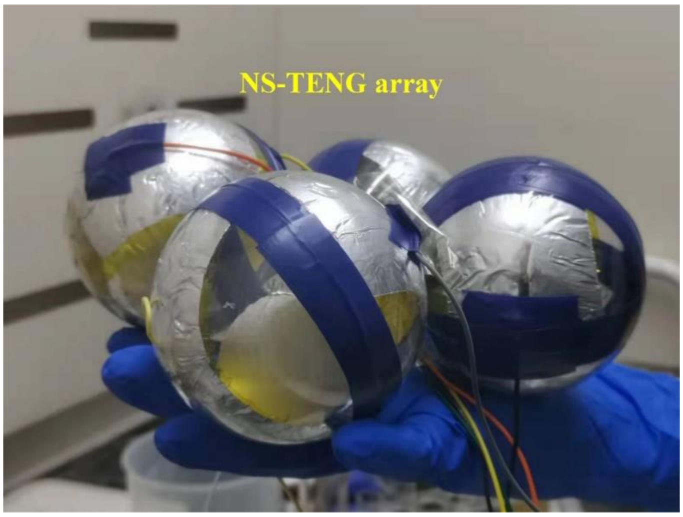

Figure S1. The photograph of light weight NS-TENG array (200g).

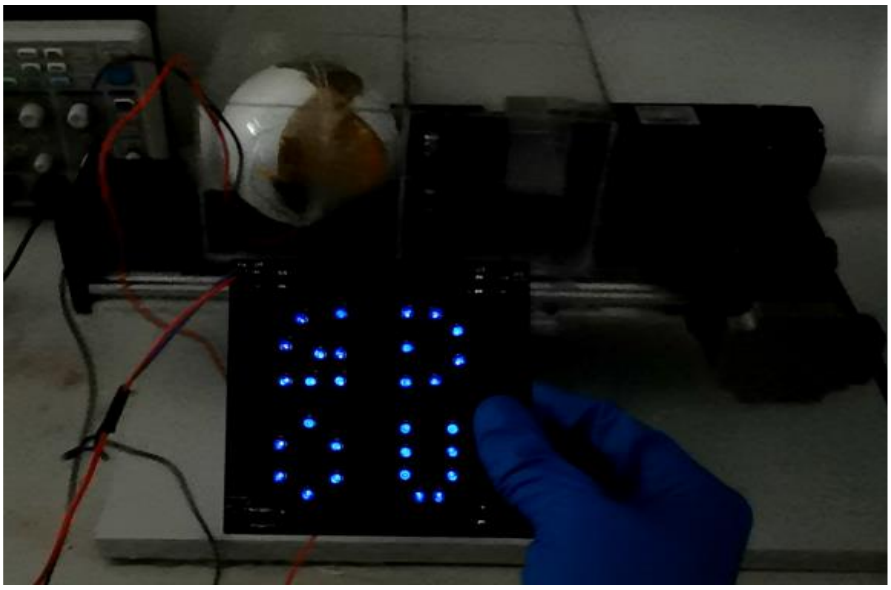

Figure S2. The photograph of single NS-TENG lighted up LED arrays (Supplementary Video S1).

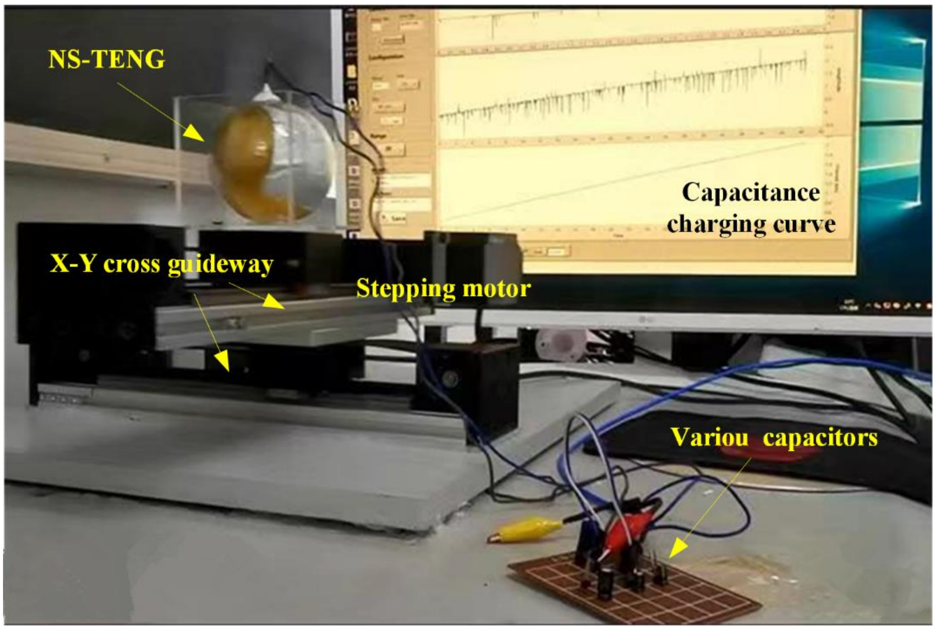

Figure S3. The photograph of the NS-TENG charging a capacitor.

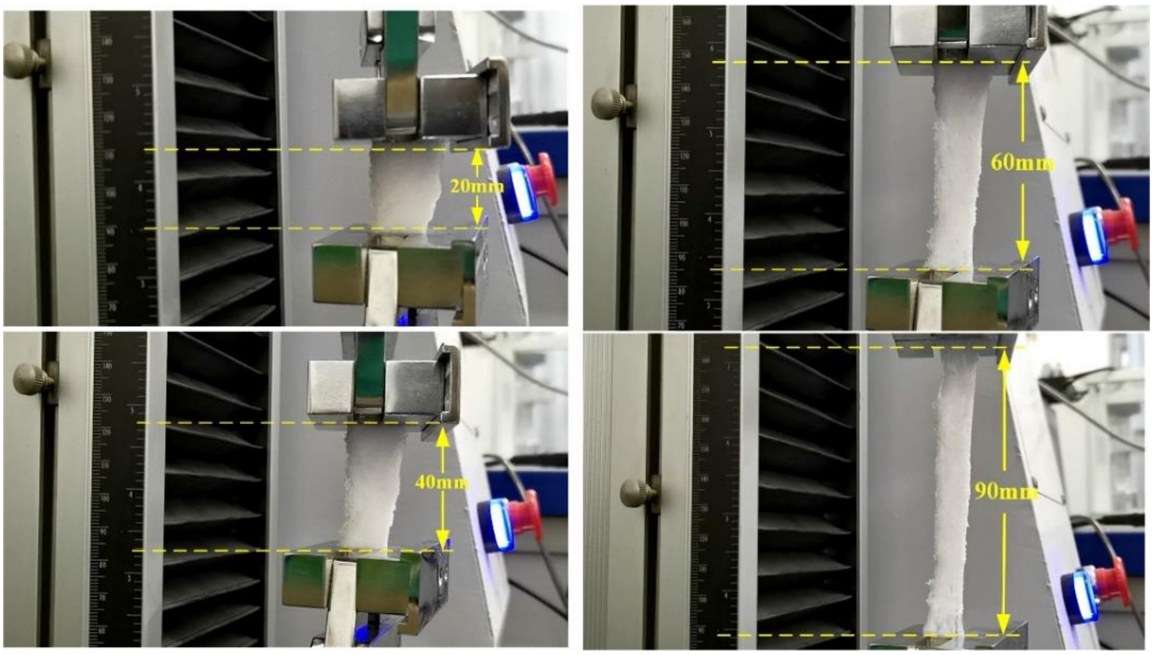

Figure S4. Photos of the NSP film (P-Eco) stretching process.

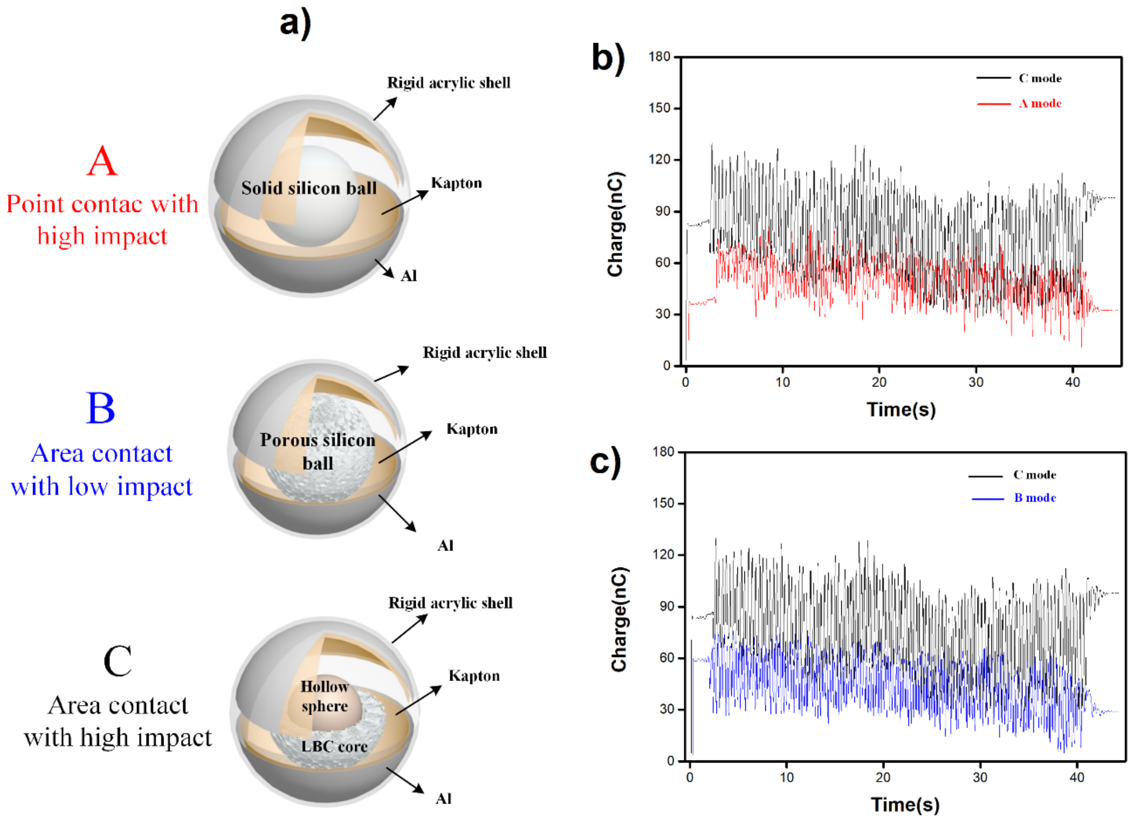

Figure S5. (**a**) schematic diagram of three different contact modes; (**b**–**c**) The comparison of output charge curves.

The effect of surface curvature on contact electrification of the same materials would have different surface energies, possibly due to the stretched or compressed surface molecules. Therefore, we analyze their surface energy states by comparing their charge curves, as shown in Figure S5. The average charge of mode C is as high as 76 nC during 40 s, much higher than the 22 nC and 32 nC of A and B, demonstrating that the energies for the C surface state mode would be shifted more efficiently considering the effects of surface energy. The higher charge levels of the C mode indicates that our soft-contact surface state stores a higher amount of charge than the others.

Table S1. The output performance comparison of rolling spherical structured TENG.

Core Material	Frequency (Hz)	Dielectric Film	Dielectric Material	Electric output performances			Ref.
				Voc	Isc	Power	
Nylon ball	1.43	Kapton	Cu	12 kV	1.2 uA	10 mW	[1]
Silicone ball (UV treated)	3	POM	Ag-Cu	1020 V	34.5 uA	5.93 mW	[2]
Silicone rubber@liquid ball	2	-	Cu	90 V	0.8 uA	45 mW	[3]
PTFE ball	1.8	Nylon	Al	120 V	5.8 uA	10.6 W/m^3	[4]
FEP ball	1.45	PTFE	Cu	-	35 uA	32.6 W/m^3	[5]
Silicone rubber ball	4.2	-	Cu	13 V	-	455 nW	[6]
PDMS ball	2	PTFE	Au	25 V	7.3 uA	18 uW/cm^3	[7]
Ecoflex@hollow sphere ball	1.5	Kapton	Al	580 V	23.5 uA	9.1 mW	This wok

Although, the electrical outputs of NS-Teng is not the highest among the above TENGs, which is comparable to spherical TENGs with multilayered structures and unique surface engineering. The cost-effective method and readily available materials result in a production cost of less than $1 for the entire NS-TENG. The porous silicon film was

prepared only using three food-grade additives: Ecoflex, cellulose and sugar. The by-product of the whole preparation process is only recyclable sugar water, realizing green and pollution-free production. It is worth mentioning, if the maximum power of spherical TENG reached 32 W/m^3 (corresponding to a high power level reported in the current literature [5]), 1 m^3 space will need thousands of 4–8 cm diameter spherical TENGs. Assuming that, the cost of single ball is about $10, the fabricating cost of 1 m^3 spherical TENG array will be at least $10,000 plus, which is much higher than the cost of energy generated by wind power ($0.5 each watt). Therefore, it is necessary to reduce the complexity of the production process while increasing the power for spherical TENG.

References

1. Wang, X.; Niu, S.; Yin, Y.; Yi, F.; You, Z.; Wang, Z.L. Triboelectric nanogenerator based on fully enclosed rolling spherical structure for harvesting low-frequency water wave energy. *Adv. Energy Mater.* **2015**, *5*, 1501467.
2. Xu, L.; Jiang, T.; Lin, P.; Shao, J.J.; He, C.; Zhong, W.; Chen, X.Y.; Wang, Z.L. Coupled triboelectric nanogenerator networks for efficient water wave energy harvesting. *ACS Nano* **2018**, *12*, 1849–1858.
3. Cheng, P.; Guo, H.; Wen, Z.; Zhang, C.; Yin, X.; Li, X.; Liu, D.; Song, W.; Sun, X.; Wang, J.; others. Largely enhanced triboelectric nanogenerator for efficient harvesting of water wave energy by soft contacted structure. *Nano Energy* **2019**, *57*, 432–439.
4. Xu, M.; Zhao, T.; Wang, C.; Zhang, S.L.; Li, Z.; Pan, X.; Wang, Z.L. High power density tower-like triboelectric nanogenerator for harvesting arbitrary directional water wave energy. *ACS nano* **2019**, *13*, 1932–1939.
5. Yang, X.; Xu, L.; Lin, P.; Zhong, W.; Bai, Y.; Luo, J.; Chen, J.; Wang, Z.L. Macroscopic self-assembly network of encapsulated high-performance triboelectric nanogenerators for water wave energy harvesting. *Nano Energy* **2019**, *60*, 404–412.
6. Wang, Y.; Matin Nazar, A.; Wang, J.; Xia, K.; Wang, D.; Ji, X.; Jiao, P. Rolling Spherical Triboelectric Nanogenerators (RS-TENG) under Low-Frequency Ocean Wave Action. *J. Mar. Sci. Eng.* **2021**, *10*, 5.
7. Chen, H.; Wang, J.; Ning, A. Optimization of a Rolling Triboelectric Nanogenerator Based on the Nano–Micro Structure for Ocean Environmental Monitoring. *ACS omega* **2021**, *6*, 21059–21065.
